# Kidney injury after lung transplantation: Long-term mortality predicted by post-operative day-7 serum creatinine and few clinical factors

**DOI:** 10.1371/journal.pone.0265002

**Published:** 2022-03-04

**Authors:** Julian Doricic, Robert Greite, Vijith Vijayan, Stephan Immenschuh, Andreas Leffler, Fabio Ius, Axel Haverich, Jens Gottlieb, Hermann Haller, Irina Scheffner, Wilfried Gwinner

**Affiliations:** 1 Department of Nephrology, Hannover Medical School, Hannover, Germany; 2 Institute of Transfusion Medicine and Transplant Engineering, Hannover Medical School, Hannover, Germany; 3 Department of Anesthesiology and Intensive Care Medicine, Hannover Medical School, Hannover, Germany; 4 Department of Cardiothoracic, Transplantation and Vascular Surgery, Hannover Medical School, Hannover, Germany; 5 Department of Respiratory Medicine, Hannover Medical School, Hannover, Germany; Medical University of Gdansk, POLAND

## Abstract

**Background:**

Acute kidney injury (AKI) after lung transplantation (LuTx) is associated with increased long-term mortality. In this prospective observational study, commonly used AKI-definitions were examined regarding prediction of long-term mortality and compared to simple use of the serum creatinine value at day 7 for patients who did not receive hemodialysis, and serum creatinine value immediately before initiation of hemodialysis (d7/preHD-sCr).

**Methods:**

185 patients with LuTx were prospectively enrolled from 2013–2014 at our center. Kidney injury was assessed within 7 days by: (1) the Kidney Disease Improving Global Outcomes criteria (KDIGO-AKI), (2) the Acute Disease Quality Initiative 16 Workgroup classification (ADQI-AKI) and (3) d7/preHD-sCr. Prediction of all-cause mortality was examined by Cox regression analysis, and clinical as well as laboratory factors for impaired kidney function post-LuTx were analyzed.

**Results:**

AKI according to KDIGO and ADQI-AKI occurred in 115 patients (62.2%) within 7 days after LuTx. Persistent ADQI-AKI, KDIGO-AKI stage 3 and higher d7/preHD-sCr were associated with higher mortality in the univariable analysis. In the multivariable analysis, d7/preHD-sCr in combination with body weight and intra- and postoperative platelet transfusions predicted mortality after LuTx with similar performance as models using KDIGO-AKI and ADQI-AKI (concordance index of 0.75 for d7/preHD-sCr vs., 0.74 and 0.73, respectively). Pre-transplant reduced renal function, diabetes, higher BMI, and intraoperative ECMO predicted higher d7/preHD-sCr (r^2^ = 0.354, p < 0.001).

**Conclusion:**

Our results confirm the importance of AKI in lung transplant patients; however, a simple and pragmatic indicator of renal function, d7/preHD-sCr, predicts long-term mortality equally reliable as more complex AKI-definitions like KDIGO and ADQI.

## Introduction

Acute kidney injury (AKI) is a common complication after lung transplantation (LuTx), with incidence rates of 39–69% [1–5] and is strongly associated with an increased risk for death [5–7]. However, reliable individual assessment of this risk is impeded by heterogenous and rather complex definitions of AKI. Applying common AKI definitions such as the Kidney Disease Improving Global Outcomes (KDIGO) definition [8] and the Acute Disease Quality Initiative 16 Workgroup definition (ADQI) [9] requires time and effort. Specifically, serum creatinine (sCr) has to be monitored prior to LuTx and on seven consecutive postoperative days to apply the KDIGO criteria. Based on the dynamic sCr changes, patients are categorized into three different AKI stages [8]. Moreover, the ADQI definition uses the KDIGO criteria but adds persistence of AKI as an attribute, which is defined as AKI presence for more than 48 hours [9]. An alternative AKI criterion proposed by KDIGO and ADQI is urine output (UO). UO needs to be measured for 24 hours and AKI can then be staged according to specific cut-offs for UO reduction [8]. However, due to unavailable data on UO, this alternative criterion has not been used in many studies [2, 5, 10–12].

Based on these considerations and on the fact that categorization of a continuous parameter like serum creatinine may decrease its informational value [13], this study examines whether a simple assessment of kidney function by the serum creatinine value at day 7 for patients who did not receive hemodialysis, and serum creatinine value immediately before initiation of hemodialysis (d7/preHD-sCr) predicts mortality equally reliable as the aforementioned measures of AKI. Furthermore, clinical and laboratory predictors of impaired post-transplant renal function are explored.

## Methods

### Study population

Patients were consecutively recruited for this study. In the period from June 2013 to December 2014, 193 adult patients received a LuTx, of which 185 patients consented to participate in the study (95.9%). Exclusion criteria were combined organ transplantations. The study was approved by the local ethics committee (no. 6895) and initiated in 2013 to examine the primary endpoint "AKI" in terms of clinical factors and biomarkers. The following results now refer to the secondary endpoint "long-term outcome". Clinical, laboratory and surgery-related factors were documented pre-, peri- and post-transplantation. Patients were followed up for 5 years post-transplant, with an annual routine consisting of clinical and laboratory parameters including renal function and immunosuppressive drug levels. The mean follow-up was 57 ± 20 months.

Minimally invasive sternum-sparing anterolateral thoracotomy performed in the transplant procedure whenever possible. The majority of patients received bilateral LuTx (n = 182, 98.4%); only 3 patients had single LuTx. Initially, all patients received a triple maintenance immunosuppression with tacrolimus, mycophenolate mofetil and steroids. Within the first year after transplantation, tacrolimus target levels were 8–12 μg/liter. Induction therapy was not used. In all patients, primary graft function (PGD) was scored at different postoperative time points after transplantation according to the International Society for Heart and Lung Transplantation guidelines and PGD grade 3 was used for analysis [14, 15].

### Renal function and AKI definitions

Renal function was determined by calculating the estimated glomerular filtration rate (eGFR) with the Chronic Kidney Disease Epidemiology Collaboration (CKD-EPI)–formula [16]. Baseline serum creatinine values were collected within 7 days before transplantation. No patient was on dialysis prior to LuTx. All patients who required renal replacement therapy (RRT) after LuTx underwent hemodialysis. Serum creatinine value at day 7 for patients who did not receive hemodialysis, and serum creatinine value immediately before initiation of hemodialysis was used for analysis (d7/preHD-sCr). The KDIGO criteria [8] were used to grade AKI as stage I (sCr increase by 1.5 to 1.9-fold from baseline or absolute sCr increase by ≥0.3 mg/dl or ≥26.5 μmol/l), stage II (sCr increase by 2.0 to 2.9-fold from baseline), or stage III (sCr increase by 3.0-fold from baseline or sCr increase by ≥4.0 mg/dl or ≥353.6 μmol/l or initiation of RRT within the first 7 days after LuTx). The ADQI definition [9] was used to differentiate between transient AKI (return of sCr below KDIGO AKI stage I within 48 hours of AKI onset) and persistent AKI (sustained sCr elevation with at least KDIGO stage I beyond 48 hours after AKI). Urine output, which is an alternative criterion in both definitions (KDIGO, ADQI), was excluded as an AKI criterion because the data was not available for all patients.

### Statistical analysis

Statistical analyses were performed with IBM SPSS statistical software version 26.0 and the rms [17] package from R Studio software 3.6.0 (R Core Team, 2020). Continuous data with normal distribution are reported as mean value ± standard deviation. Data with non-normal distribution are reported with median and interquartiles. The Kolmogorov-Smirnov test was used to identify variables with and without normal distribution. Group comparisons were made with the student’s t-test and the Mann–Whitney U-test accordingly. To examine cut-points of continuous parameters in the context of survival analyses, we used R Studio package ’CutpointsOEHR’ [18]. Categorical variables are presented as numbers and percentages and comparisons were made using Fisher’s exact test and chi-square test. Kaplan-Meier analysis and log-rank test were used to describe patient survival for all causes of mortality.

For the Cox regression analyses, linearity of continuous variables was verified by categorizing the variables and comparing the ß-coefficients from the univariate Cox regression. Variables with a p-value <0.05 in the univariate analysis and further variables that were deemed biologically relevant were included in the multivariable modeling, which was performed by stepwise backward selection (p-value threshold <0.15). The bootstrapping procedures in R software were used to validate the models. The Harrell’s concordance index was used to report the performance of the models [19]. Kaplan-Meier curves were constructed from multivariate Cox model for 4 separate risk groups ("very low" to "high") using cut points on the prognostic index determined by the Cox method (cut points: 16th, 50th, and 84th percentiles of the prognostic index) [20, 21]. In the absence of any significant difference and with identical Kaplan-Meier curves, the two risk groups “very low” and “low” were summarized into one group "low" for better clarity. Multivariable linear regression analysis was applied to assess the effect of pre-and perioperative factors on d7/preHD-sCr after LuTx, using backward selection and a cut-off p value of <0.2. Unstandardized predicted serum creatinine values were calculated using the regression equation based on the unstandardized regression coefficients. Differences with p <0.05 were considered as significant.

## Results

### 1. Study population

Patient characteristics, pre- and postoperative factors of the entire study population and the subgroups with and without AKI stage I-III according to KDIGO are shown in Table 1. Leading indications for LuTx were idiopathic pulmonary fibrosis (33.0%), chronic obstructive pulmonary disease (23.8%) and cystic fibrosis (20.0%). Mean age at transplantation was 48y (± 12yrs). Sex distribution between patients with and without AKI was comparable. Patients with AKI post LuTx were younger and more frequently had diabetes and better kidney function before LuTx. Surgery time and post-operative intensive care unit treatment was longer and more packed red blood cells (pRBC) were given during surgery to the patients, that later developed AKI. Primary graft dysfunction of grade 3, which has been shown predictive for survival after lung transplantation, was only numerically more prevalent in patients with AKI.

**Table 1:** Patient characteristics and peri- and postoperative data of the study cohort.

	All pts.	no AKI	AKI	p-value
	n = 185	n = 70	n = 115	
**Recipient**				
Age (years), median (IQR)	50 (40.5–58.0)	52.0 (45.0–59.0)	48.0 (36.0–57.0)	0.013
Male/female, n (%)	91 / 94 (49.2/50.8)	35 / 35 (50.0/50.0)	56 /59 (48.7/51.3)	0.863
**Indication for LuTx, *n (%)***				
Chronic obstructive pulmonary disease, emphysema	44 (23.8)	18 (25.7)	26 (22.6)	0.630
Idiopathic pulmonary fibrosis	61 (33.0)	25 (35.7)	36 (31.3)	0.536
Cystic fibrosis	37 (20.0)	8 (11.4)	29 (25.2)	0.022
Sarcoidosis	11 (5.9)	4 (5.7)	7 (6.1)	0.917
Bronchiolitis obliterans syndrome*	7 (3.8)	0 (0.0)	7 (6.1)	0.045
Pulmonary hypertension	5 (2.7)	3 (4.3)	2 (1.7)	0.300
Alpha-1 anti-trypsin deficiency	6 (3.2)	6 (8.6)	0 (0.0)	0.003
Other indications	14 (7.6)	6 (8.6)	8 (7.0)	0.687
***Co-morbidities***				
Arterial hypertension, n (%)	56 (30.3)	22 (31.4)	34 (29.6)	0.789
Diabetes, n (%)	37 (20.1)	5 (7.2)	32 (27.8)	0.001
History of smoking, n (%)	64 (34.6)	32 (46.4)	32 (27.8)	0.011
Body weight at LuTx (kg), mean ± SD	65.2 ± 13.9	66.4 ± 13.3	64.6 ± 14.3	0.384
Body mass index at Tx (kg/m^2^), median (IQR)	22.3 (19.1–25.1.)	23.0 (19.5–25.1)	22.0 (18.9–25.2)	0.500
**Pre-transplant factors**				
Serum creatinine before LuTx (μmol/l), median (IQR)	61.5 (49.3–74.0)	65.0 (54.0–82.0)	58.0 (47.0–72.0)	0.011
eGFR before LuTx (ml/min), median (IQR)	105.3 (93.5–116.9)	100.0 (87.4–109.4)	107.6 (97.3–124.9)	0.001
Lung Allocation Score**, median (IQR)	36.5 (33.5–42.6)	36.0 (32.8–40.6)	36.8 (33.8–43.5)	0.194
FEV 1 (%), median (IQR)	24.0 (17.0–42.0)	25.0 (17.0–47.5)	24.0 (18.0–36.0)	0.717
FVC (%), median (IQR)	40.0 (32.0–50.0)	40.0 (32.0–54.5)	40.0 (31.0–50.0)	0.471
Donor gender (%; male/female)	51.4 / 48.6	45.7 / 54.3	54.8 / 45.2	0.231
Serum C-reactive protein (mg/l), median (IQR)	6.0 (2.0–18.9)	6.0 (2.0–14.5)	7.0 (2.0–22.5)	0.384
**Peri-transplant factors**				
Operation time (min), median (IQR)	304.0 (270.0.– 353.0)	287.0 (248.5–323.0)	311.0 (279.0–359.0)	0.001
Ischemia time first lung side (min), mean ± SD	407 ± 108	392 ± 110	416 ± 105	0.151
Ischemia time second lung side (min), mean ± SD	536 ± 116	511 ± 115	551 ± 114	0.031
ECMO preoperative, n (%)	10 (5.4)	2 (2.9)	8 (7.0)	0.323
ECMO intraoperative, n (%)	40 (21.6)	12 (17.4)	28 (24.3)	0.268
Intraoperative transfused pRBC, median (IQR) /(min-max)	2.0 (2.0–3.0)/ (0.0–21.0)	2.0 (0.0–2.5)/ (0.0–8.0)	2.0 (0.0–4.0)/ (0.0–21.0)	0.031
Intraoperative transfused platelets, median (IQR) /(min-max)	0.0 (0.0–0.75)/ (0.0–4.0)	0.0 (0.0–0.0)/ (0.0–2.0)	0.0 (0.0–1.0)/ (0.0–4.0)	0.177
Intraoperative transfused FFP, median (IQR)/(min-max)	3.0 (0.0–4.0)/ (0.0–16.0)	4.0 (0.0–4.0)/ (0.0–10.0)	2.0 (0.0–4.0)/ (0.0–16.0)	0.593
**Immunosuppressive therapy**				
Averaged tacrolimus levels from day 1 to 7 (μg/l), median (IQR)	5.4 (4.0–6.7)	5.3 (4.3–6.8)	5.4 (4.1–6.9)	0.981
**Post-transplant-related factors**				
PGD Grade 3 any time 0-72h, n (%)	17 (9.2)	3 (4.3)	14 (12.2)	0.113
d7/preHD-sCr (μmol/l), median (IQR)	72.5 (54.0–96.5)	63.0 (51.0–74.5)	80.0 (56.8–111.3)	0.001
eGFR at day 7 (ml/min), median (IQR)	97.6 (71.4–112.2)	100.9 (87.3–112.5)	88.9 (60.2–111.5)	0.008
KDIGO-AKI stage 1, n (%)		-	46 (40.0)	
KDIGO-AKI stage 2, n (%)		-	51 (44.3)	
KDIGO-AKI stage 3, n (%)		-	18 (15.7)	
Transient ADQI-AKI, n (%)		-	55 (47.8)	
Persistent ADQI-AKI, n (%)		-	60 (52.2)	
Dialysis post Tx during first 7 days, n (%)		-	4 (3.5)	
Re-operation, n (%)	33 (17.8)	9 (12.9)	24 (20.9)	0.167
Postoperative transfused pRBC, median (IQR)/(min-max)	2.0 (0.0–3.0)/ (0.0–47.0)	2.0 (0.0–2.0)/ (0.0–8.0)	2.0 (0.0–4.0)/ (0.0–47.0)	0.200
Postoperative transfused platelets, median (IQR)/(min-max)	0.0 (0.0–1.0)/ (0.0–17.0)	0.0 (0.0–0.0)/ (0.0–5.0)	0.0 (0.0–1.0)/ (0.0–17.0)	0.074
Postoperative transfused FFP, median (IQR)/(min-max)	1.0 (0.0–2.0)/ (0.0–25.0)	0.0 (0.0–2.0)/ (0.0–7.0)	2.0 (0.0–3.0)/ (0.0–25.0)	0.074
Post Tx hospital stay (days), median (IQR)	23 (21–32)	23 (21–29)	23 (21–33)	0.176
Intensive care unit treatment (days), median (IQR)	2 (1–4)	2 (1–3)	2 (1–4)	0.058

AKI is defined according to the KDIGO criteria, including stages 1–3. Normally distributed variables are presented using mean and standard deviation (SD), non-normally distributed variables by median and interquartile ranges.

*bronchiolitis obliterans syndrome refers to a previous lung transplantation

**Lung Allocation Score: the LAS predicts the survival likelihood within the following year on the waiting list and the survival likelihood one year after lung transplantation based on patient-related variables. Patients with higher LAS scores are prioritized for lung transplantation because they are expected to have a higher likelihood of survival [22].

Abbreviations: d7/preHD-sCr, serum creatinine value at day 7 for patients who did not receive hemodialysis, and serum creatinine value immediately before initiation of hemodialysis; ADQI, Acute Disease Quality Initiative; AKI, acute kidney injury; ECMO, extracorporeal membrane oxygenation; eGFR, estimated glomerular filtration rate; FEV1, forced expiratory volume in one second; FFP, fresh frozen plasma; FVC, forced vital capacity; IQR, interquartile ranges; KDIGO, Kidney Disease Improving Global Outcomes; LuTx, lung transplantation; max, maximum; min, minimum; PGD, primary graft dysfunction; pRBC, packed red blood cells; Tx, transplantation.

### 2. Incidence and characteristics of AKI

**Fig 1:**
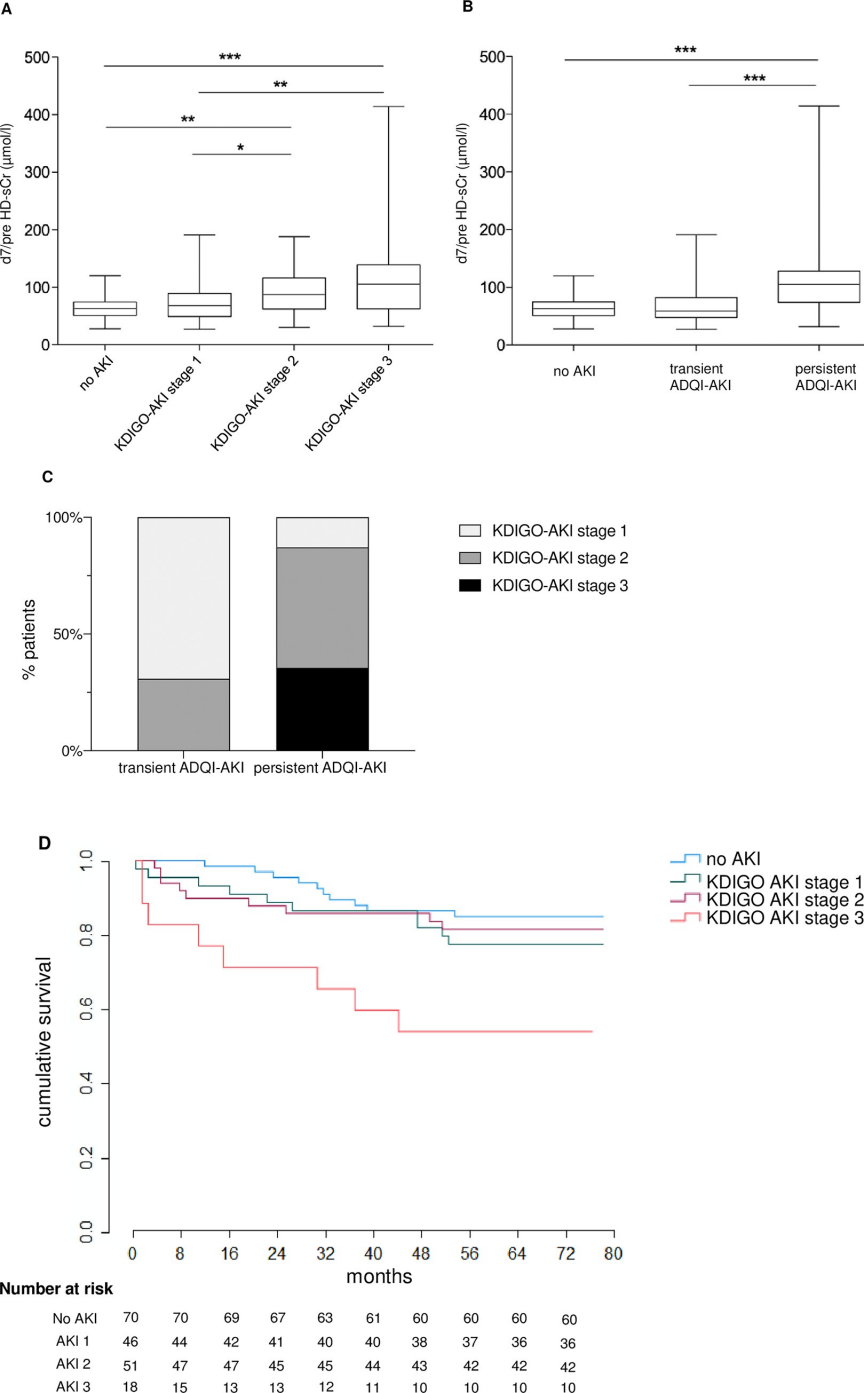
A-C Serum creatinine values at day 7 after LuTx in patients without AKI and with AKI. According to the KDIGO-AKI stages (A) and the ADQI-AKI grading (B) and distribution of the AKI stages (C). Boxes and whiskers represent medians, lower and upper quartiles, and the extreme values. *p < = 0.05; **p < = 0.01; ***p < = 0.001. (D) Long-term mortality after LuTx. Kaplan-Meier curves were stratified by stages of AKI according to the KDIGO classification. No AKI vs. KDIGO-AKI stage 1, log rank p = 0.311; No AKI vs. KDIGO-AKI stage 2, log rank p = 0.573; No AKI vs. KDIGO-AKI stage 3, log rank p = 0.002; KDIGO-AKI 1 vs. KDIGO-AKI stage 2, log rank p = 0.176; KDIGO-AKI stage 1 vs. KDIGO-AKI stage 3, log rank p = 0.043; KDIGO-AKI stage 2 vs. KDIGO-AKI stage 3, log rank p = 0.017.

Of the 185 patients, 115 (62.2%) developed KDIGO-AKI, mostly stage 1 (40.0%) and stage 2 (44.3%). Sixty patients with AKI (52.2%) did not recover renal function within 48 hours thus presenting with persistent ADQI-AKI (Table 1). Fig 1 shows the serum creatinine levels in the different AKI stages. Median d7/preHD-sCr was comparable between no AKI and KDIGO-AKI stage 1 (Fig 1A) and between no AKI and transient ADQI-AKI (Fig 1B). Patients with transient AKI had KDIGO-AKI stage 1 in 69% and the remaining patients had KDIGO-AKI stage 2. Patients with persistent ADQI-AKI had mostly KDIGO-AKI stage 2 (56.7%) and stage 3 (30.0%), with 4 patients requiring dialysis (Fig 1C).

### 3. AKI and patient long-term survival

In the post-transplant follow-up period of 57 ±20 months, one patient was lost in the -to-follow-up and 36 (19.5%) patients died. Leading causes of death were infections (n = 16; 44.4%), chronic lung allograft dysfunction (n = 12; 33.3%) such as bronchiolitis obliterans syndrome and restrictive allograft syndrome, and malignancies in 4 patients. One patient each died from sudden cardiac death, acute graft failure, hemoptysis and ileus. Five of the 16 infectious deaths occurred in the early post-transplant course within the first 90 days. All infectious deaths except one were in patients with AKI. Overall, in the patients without AKI 9 deaths occurred, whereas the remaining 27 deaths were observed in patients with AKI, with the worst survival in KDIGO-AKI stage 3 (Fig 1D). Notably, three of the four patients who required dialysis within the first 7 days after transplantation died.

In univariable Cox analyses (Table 2), KDIGO-AKI stage 3, persistent ADQI-AKI and higher d7/preHD-sCr were associated with reduced survival. Regarding the d7/preHD-sCr, there was a linear relation with mortality, without discernible cut-off value. Further factors associated with reduced survival were higher body weight and several variables relating to a more complex course of surgery and postoperative treatment such as operating time, re-operation, ECMO treatment, length of stay in the ICU and ward, substitution of blood components and primary graft dysfunction at 72 hours after transplantation. The four patients with dialysis requirement until day 7 had the longest operation times (median 374.5 min), and more transfusions of pRBC and FFP (median 12.5 and 9.5) intraoperatively.

**Table 2:** Hazard ratios of potential pre-, peri-, and postoperative factors for death in univariable Cox regression analysis.

	HR	CI-95%	p value
Age (years)	1.003	0.976–1.030	0.856
Male sex	1.699	0.874–3.303	0.118
**Indication for LuTx**			
Chronic obstructive pulmonary disease, emphysema	0.500	0.119–2.093	0.343
Cystic fibrosis	0.743	0.186–2.971	0.675
Idiopathic fibrosis	1.173	0.340–4.052	0.801
Sarcoidosis	1.383	0.279–6.853	0.691
Bronchiolitis obliterans syndrome*	1.502	0.251–8.990	0.656
Pulmonary hypertension	0.954	0.099–9.175	0.968
Alpha-1 antitrypsin deficiency	1.610	0.269–9.639	0.602
Diabetes	1.492	0.722–3.083	0.279
Body weight (kg)	1.026	1.002–1.050	0.030
Operation time (min)	1.006	1.002–1.010	0.007
**No. of transfused pRBC intraoperative**			
3–7	1.510	0.727–3.136	0.269
> 8	4.688	1.858–11.828	0.001
**No. of transfused FFP intraoperative**			
> 5	2.007	0.992–4.063	0.053
**No. of transfused platelets intraoperative**			
> 2	3.097	1.592–6.024	0.001
ECMO intraoperative	2.246	1.143–4.413	0.019
Re-operation	3.078	1.566–6.047	0.001
eGFR before LuTx (ml/min)	0.994	0.979–1.010	0.449
Serum-creatinine before LuTx (μmol/l)	1.010	0.995–1.025	0.188
d7/preHD-sCr (μmol/l)	1.011	1.006–1.016	< 0.0001
**AKI stages**			
KDIGO-AKI stage 1	1.564	0.651–3.759	0.317
KDIGO-AKI stage 2	1.287	0.523–3.167	0.583
KDIGO-AKI stage 3	3.978	1.569–10.086	0.004
Transient ADQI-AKI	0.994	0.392–2.518	0.989
Persistent ADQI-AKI	2.587	1.203–5.566	0.015
Dialysis post Tx during first 7 days	8.846	3.842–20.367	< 0.0001
**Primary graft dysfunction**			
Grade 3 at 48h or 72	0.892	0.214–3.713	0.875
**No. of transfused FFP postoperative**			
3–5	2.532	1.152–5.565	0.021
≥ 6	6.093	2.679–13.861	< 0.0001
**No. of transfused pRBC postoperative**			
1–5	1.191	0.521–2.721	0.678
6–10	1.766	0.468–6.660	0.401
> 10	5.359	1.940–14.805	0.001
**No. of transfused platelets postoperative**			
> 1	2.905	1.521–5.548	0.001
**Post Tx hospital stay (days)**			
≥ 28 days	2.050	1.073–3.914	0.030
**Intensive care unit stay (days)**			
2 days	1.463	0.530–4.034	0.462
> 3 days	3.421	1.522–7.687	0.003

For the variables ‘packed red blood cell infusion intraoperatively’ and ‘postoperative fresh frozen plasma’, 0–2 units represent the reference category with a HR of 1.0, the lowest nominal category represents the reference category. Lower and upper limits of the 95% confidence interval (CI-95%) of the hazard ratio (HR) are shown.

Abbreviations: d7/preHD-sCr, serum creatinine value at day 7 for patients who did not receive hemodialysis, and serum creatinine value immediately before initiation of hemodialysis; ADQI, Acute Disease Quality Initiative; AKI, acute kidney injury; ECMO, extracorporeal membrane oxygenation; FFP, fresh frozen plasma; HR, hazard ratio; KDIGO, Kidney Disease Improving Global Outcomes; LuTx, lung transplantation; PGD, primary graft dysfunction; pRBC, packed red blood cells; Tx; transplantation.

*bronchiolitis obliterans syndrome refers to a previous lung transplantation.

Based on these univariable results (Table 2), multivariable models with the different AKI definitions and d7/preHD-sCr were created. These results are summarized in Table 3A–3C. The predictive performance was similar for the two AKI definitions and d7/preHD-sCr, with a concordance index of 0.75 for d7/preHD-sCr, 0.74 for KDIGO-AKI and 0.73 for ADQI-AKI and, after 200-fold bootstrapping, 0.74 for d7/preHD-sCr, and 0.70 for the other two models. The variables used in the three models were similar.

**Table 3:** Long-term prediction of all-cause mortality by multivariable Cox regression models with two AKI definitions (A and B) and d7/preHD-sCr (C).

A			
**KDIGO-AKI**			
**Concordance index 0.74 (0.70)**			
	HR	p	CI-95%
Body weight (kg)	1.035	0.005	1.010–1.059
>2 transfused platelets intraoperative	2.535	0.010	1.246–5.157
>1 transfused platelet postoperative	2.522	0.010	1.252–5.078
KDIGO-AKI stage 1	1.570	0.314	0.652–3.778
KDIGO-AKI stage 2	0.892	0.809	0.352–2.259
KDIGO-AKI stage 3	3.504	0.014	1.290–9.517
B			
**ADQI-AKI**			
**Concordance index 0.73 (0.70)**			
	HR	p	CI-95%
Body weight (kg)	1.028	0.017	1.005–1.052
>2 transfused platelets intraoperative	2.022	0.052	0.994–4.115
3–5 transfused FFP postoperative	1.999	0.088	0.902–4.429
≥ 6 transfused FFP postoperative	4.726	0.001	1.875–11.915
Transient ADQI-AKI	0.760	0.582	0.287–2.017
Persistent ADQI-AKI	2.022	0.084	0.909–4.498
C			
**d7/preHD-sCr**			
**Concordance index 0.75 (0.73)**			
	HR	p	CI-95%
Body weight (kg)	1.030	0.024	1.004–1–057
>2 transfused platelets intraoperative	2.131	0.036	1.050–4.324
>1 transfused platelet postoperative	2.248	0.020	1.138–4.442
d7/preHD-sCr (μmol/L)	1.011	< 0.0001	1.005–1.017

Variables for the models were selected as described in the Methods and according to the univariable Cox analyses (Table 2). Table 3A–3C depict only the variables that were retained in multivariable models. Concordance index value in brackets indicates the value after 200-fold bootstrapping.

Abbreviations: ADQI, Acute Disease Quality Initiative; AKI, acute kidney injury; CI-95%, upper and lower limits of the 95% confidence interval; d7/preHD-sCr, serum creatinine value at day 7 for patients who did not receive hemodialysis, and serum creatinine value immediately before initiation of hemodialysis; FFP, fresh frozen plasma; HR, hazard ratio; KDIGO, Kidney Disease Improving Global Outcomes.

**Fig 2:**
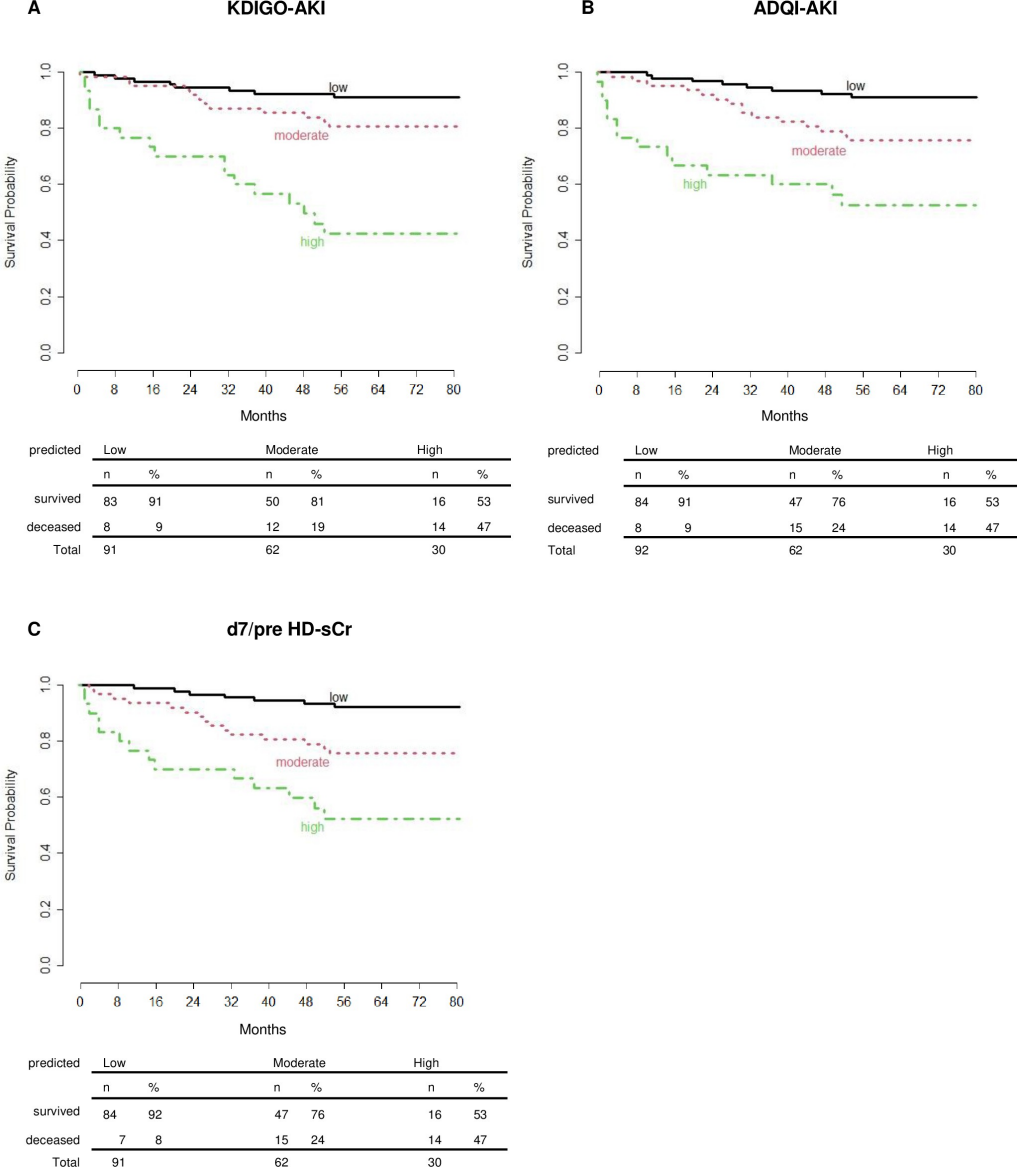
A-C. Calibration of the multivariable Cox models with KDIGO-AKI criteria (A), ADQI-AKI grading (B) and d7/preHD-sCr (C). Kaplan-Meier curves are shown for three separate risk groups ("low" to "high") as described in Methods. The table below shows the events observed in the three risk groups.

The calibration of the three models was comparable, with similar proportions of patients assigned to low, moderate and high mortality risk according to the model’s prognostic index (Fig 2). We also tested the d7/preHD-sCr model including dialysis as an additional variable and the results are shown in S1 Table. The performance of this model and the risk factors remained unchanged. An additional model using the eGFR calculated from d7/preHD-sCr showed a concordance index of 0.74/0.71 (without/with bootstrapping) and thus had no advantage. PGD grade 3 was not retained as a significant factor in the models.

**Fig 3:**
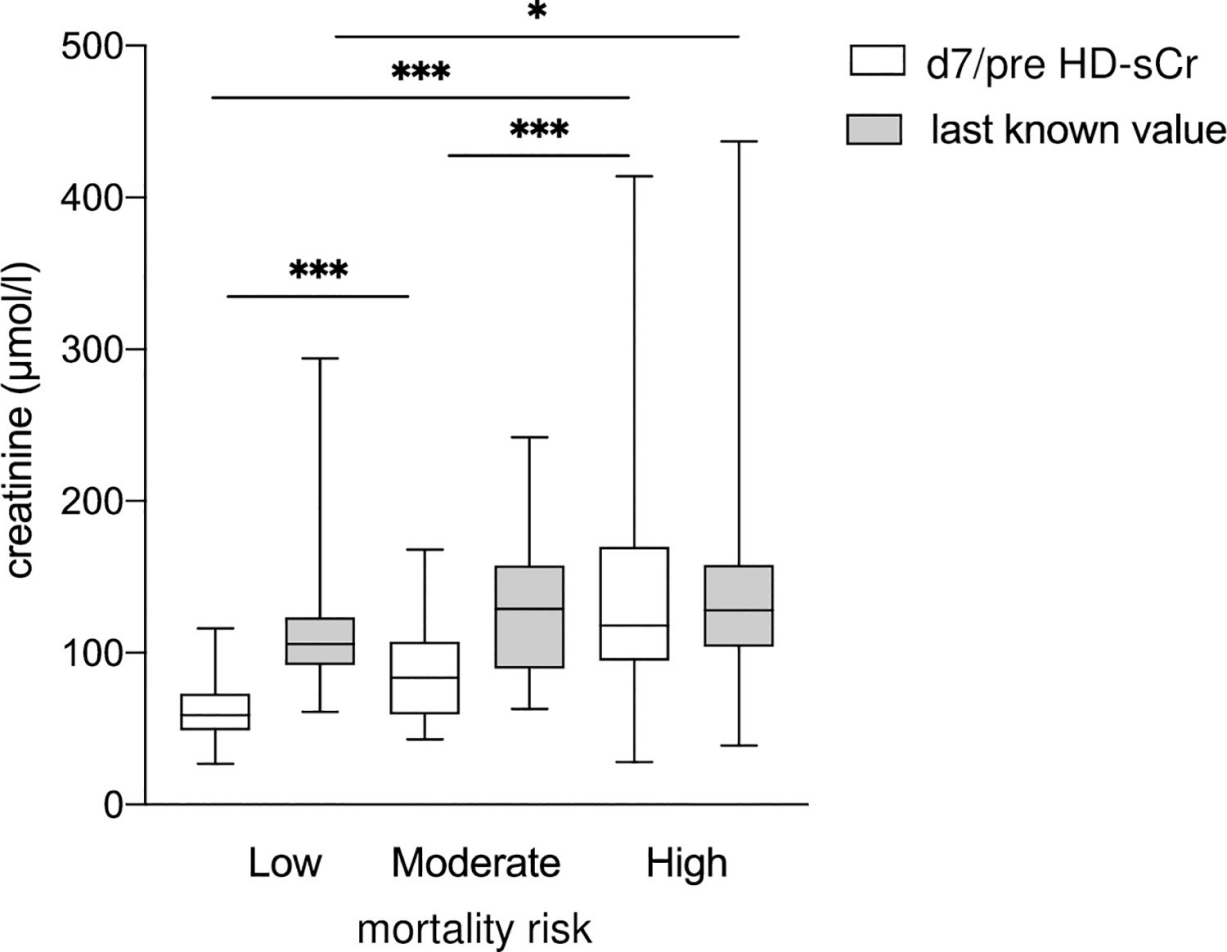
Serum creatinine values at the last follow-up in the three risk groups assigned by the d7/preHD-sCr multivariable model. For comparison, the d7/preHD-sCr value is also depicted for the three risk groups. For patients with dialysis, the serum creatinine level at initiation of renal replacement therapy was used. Boxes and whiskers represent medians, lower and upper quartiles, and the extreme values. *p < = 0.05; **p < = 0.01; ***p < = 0.001.

In a post-hoc analysis of the long-term course after discharge from the hospital, renal function was examined in the patients assigned to low, moderate and high mortality risk. The four patients who required dialysis within the first 7 days were in the high-risk group. Fig 3 shows the last known sCr value during routine follow-up over 57 ±20 months in the three risk groups defined by the d7/preHD-sCr prediction model. sCr values at last follow-up were higher in all three groups, but highest in patients with moderate and high mortality risk.

### 4. Prediction of d7/preHD-sCr

**Fig 4:**
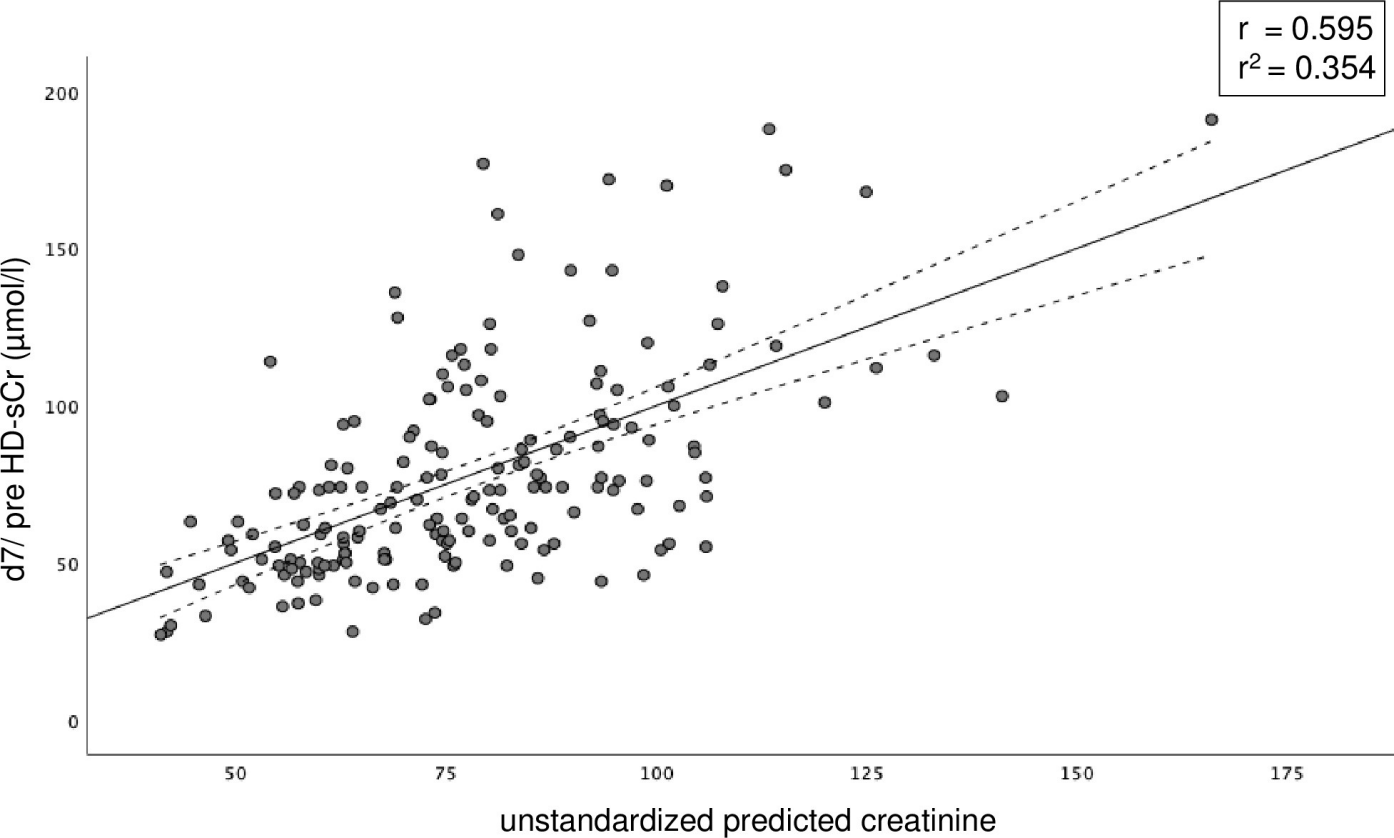
Predicted and observed d7/preHD-sCr after LuTx. Results were obtained by the multivariable regression model with pretransplant serum creatinine, diabetes, BMI and need of intraoperative ECMO as factors. Individual patient values and the regression line with the 95% confidence interval boundaries are shown.

To estimate the relationship between d7/preHD-sCr and several pre- and perioperative factors, univariable and multivariable linear regression analyses were performed (Table 4). In the multivariable analysis, pretransplant renal function, diabetes, higher BMI and need of intraoperative ECMO were retained as significant predictors of d7/preHD-sCr. Fig 4 illustrates the relation between individually observed d7/preHD-sCr levels and the sCr values predicted by the multivariable model, explaining more than a third of variation in d7/preHD-sCr (r^2^ = 0.354).

**Table 4:** Predictors of the d7/preHD-sCr after LuTx.

	univariable linear analysis				multivariable linear analysis			
	B	Beta	CI-95% for B	p value	B	Beta	CI-95% for B	p value
Age	0.385	0.170	-0.166–0.936	0.102	0.139	0.050	-0.292–0.570	0.526
Sex (male)	-11.923	-0.131	-25.158–1.312	0.077	-3.957	-0.059	-13.329–5.414	0.406
Operation time (min)	0.123	0.180	0.024–0.222	0.015	0.017	0.033	-0.070–0.104	0.707
Serum-creatinine before LuTx (μmol/l)	0.678	0.293	0.354–1.003	< 0.0001	**0.801**	**0.473**	**0.590–1.011**	**< 0.0001**
Serum C-reactive protein before LuTx (mg/l)	0.162	0.149	0.005–0.320	0.044	0.082	0.102	-0.016–0.180	0.102
Body weight (kg)	0.211	0.064	-0.269–0.690	0.387				
Body Mass Index (kg/m^2^)	1.441	0.116	-0.363–3.244	0.117	**1.217**	**0.133**	**0.054–2.379**	**0.040**
Diabetes	15.531	0.136	-1.020–32.082	0.066	**24.244**	**0.292**	**14.053–34.435**	**< 0.0001**
History of smoking	-10.180	-0.106	-24.172–3.811	0.153	-2.74	-0.039	-12.425–6.945	0.577
No. of transfused platelets intraoperative	9.240	0.174	1.564–16.917	0.019	0.146	0.004	-7.596–7.889	0.97
No. of transfused FFP intraoperative	1.862	0.118	-0.432–4.156	0.111	-1.059	-0.088	-2.627–0.508	0.184
No. of transfused pRBC intraoperative	3.032	0.209	0.949–5.115	0.005	1.133	0.099	-1.011–3.277	0.299
ECMO preoperative	20.747	0.103	-8.614–50.109	0.165	-11.815	-0.077	-33.153–9.524	0.276
ECMO intraoperative	19.853	0.179	3.882–35.824	0.015	**10.106**	**0.123**	**0.085–20.127**	**0.048**
Ischemia time first side (min)	0.023	0.054	-0.041–0.086	0.476				
Ischemia time second side (min)	0.030	0.074	-0.030–0.089	0.331				
Lung Allocation Score*	0.199	0.055	-0.337–0.735	0.464				
FEV 1 (%)	0.148	0.059	-0.221–0.517	0.430				
FVC (%)	0.241	0.089	-0.154–0.637	0.230				
Hematocrit preoperative (%)	-0.514	-0.061	-1.744–0.717	0.411				
Hemoglobin preoperative (g/dl)	-0.625	-0.028	-3.913–2.664	0.708				
Cystic fibrosis	0.782	0.007	-15.870–17.434	0.926				

For all variables, the unstandardized regression coefficient B and the standardized coefficient beta are shown. The multivariable linear regression model had a regression coefficient of 0.60 (r^2^ = 0.35).

*Lung Allocation Score: the LAS predicts the survival likelihood within the following year on the waiting list and the survival likelihood one year after lung transplantation based on patient-related variables. Patients with higher LAS scores are prioritized for lung transplantation because they are expected to have a higher likelihood of survival [22].

Abbreviations: CI-95%, 95% confidence interval; ECMO, extracorporeal membrane oxygenation; FEV1, forced expiratory volume in one second; FFP, fresh frozen plasma; FVC, forced vital capacity; LuTx, lung transplantation; pRBC, packed red blood cells.

## Discussion

This study underlines the importance of kidney injury in patient outcomes after lung transplantation. Similar to previous studies we show that higher degree of AKI early after LuTx is associated with death [4, 5]. In addition, end stage renal failure and chronic renal function impairment in the long-term were more common in patients with severe AKI early after LuTx.

Unlike previous studies which used more complex definitions of AKI in the prediction of mortality risk, this study suggests that simple use of d7/preHD-sCr is equally reliable. Regular laboratory monitoring after major surgery is essential and use of this information should be as simple as possible. This includes close monitoring of sCr to detect renal dysfunction and initiate further diagnostics and therapy. Mortality risk prediction based on KDIGO and ADQI criteria is established, but any reliable simplification is an improvement. There is good rationale to use the serum creatinine. First, categorization of variables that are continuous is rarely sensible because information is lost, particularly with broad categories [13]. Second, calculation of AKI stages is more cumbersome and may be prone to error, requiring daily measurement and on-going comparison of the actual value with baseline serum creatinine before LuTx and urine output monitoring. Notably, although the established definitions suggest urine output as an alternative criterion for AKI, many studies did not consider urine output for AKI assessment, mainly for practical reasons of availability [2, 5, 10–12]. This suggests that parameters for assessment of AKI should be kept as simple as possible. A further, principal aspect is that the d7/preHD-sCr reflects pre-existing chronic renal function impairment and acute renal function decline, as patients may not fulfill the KDIGO or ADQI criteria but nevertheless have impaired renal function and thus an increased risk of mortality.

Fairly accurate estimation of the mortality risk was shown with all parameters of kidney injury, d7/preHD-sCr, the KDIGO-AKI and the ADQI grading, together with a few additional clinical factors. The multivariable models all had similar concordance indices and differentiation of mortality risks as illustrated by the calibration studies. The model with d7/preHD-sCr included body weight and need of intra-operative and post-operative platelet infusions. These risk factors were also included in the other two models. Using the eGFR at day 7 instead of d7/preHD-sCr was not advantageous in the model, probably because of the inaccuracy of eGFR calculations in critically ill patients as reported in liver transplant patients [23, 24].

The incidence of AKI is high early after LuTx, affecting one to two thirds of patients [1–5] and in our study 61% of patients. This broad range perhaps reflects different populations at risk, varying surgical and medical practice and use of different definitions of AKI. Likewise, the association of AKI with death was reported highly variable among different studies [1–6, 25, 26]. Severity of AKI was important for the mortality risk in our study, regardless of the AKI definition used. A strong link between severe AKI and death was also reported by others, particularly in patients with AKI who require temporary dialysis or who are on incident chronic dialysis treatment [6, 27–29]. This is in line with a previous study that identified severe AKI with renal replacement therapy after non-cardiac surgery as an independent risk factor for severe infections [30]. In our study, four patients required dialysis before day 7 and further six patients developed dialysis dependency within three months post-transplantation. Of these patients, all remained on dialysis and seven of them died. Infections were the leading cause of death in the early post-transplant period and even more so in the long-term and were almost exclusively prevalent in patients with AKI. In this regard, AKI has been described as an impaired immune state associated with dysfunctional monocyte cytokine production, predisposing to infections [31]. In the AKI group, sCr levels before transplantation were lower compared with the group without AKI. A similar observation was reported by others and appears to be related to the subgroup of patients with cystic fibrosis [1, 3, 11]. In our study, CF patients had a lower median pre-transplant sCr of 47.0 compared with patients without AKI. Hyperfiltration due to diabetes may be one explanation for the lower sCr in these patients, as 19 out of 37 had diabetes.

Apart from AKI, particularly factors that indicate a more complicated transplantation and post-transplantation course such as operation time, a greater need of substituting blood components, and post-surgical complications like re-operation showed associations with mortality. Platelet and plasma infusions were retained in the multivariable analyses, suggesting an independent effect on patient survival. Platelet transfusions were also a risk factor for mortality in patients with liver transplantation [32] and with lung transplantation [33].

More complex surgical courses and postoperative complications in turn can precipitate AKI, which would be in this case a surrogate of a more severe transplant course. On the other hand, an independent effect of AKI on long-term survival is indicated by various studies. AKI was linked to the development of chronic kidney disease in several study populations [34, 35] In turn, chronic kidney disease is an independent factor for mortality, besides classical risk factors like cardiovascular disease and diabetes [36]. Our post-hoc analysis emphasizes that patients after LuTx are at high risk of developing or aggravating clinically relevant chronic renal function impairment. Declining renal function was observed in all three risk groups in the long-term, most notably in the moderate- and high-risk group. This is probably due, in part, to the extensive exposure to calcineurin inhibitors [37–39].

Given the clinical relevance of AKI, identifying patients at risk is important. Pre-existing diabetes mellitus, need of intraoperative ECMO, higher BMI and impaired renal function prior to LuTx allowed prediction of higher d7/preHD-sCr. These factors were risk factors for AKI in previous studies [4, 6]. As there are no specific therapies to treat AKI, identification of patients at higher risk is crucial to modify treatment strategy, e.g. avoidance of nephrotoxic drugs and radiocontrast media, maintaining adequate cardiovascular function and treatment of relevant infections [40].

Our study has several limitations. First, this is a single-center study with a moderately-sized study population. Second, like in many other studies [2, 5, 10, 11], urine output was too difficult to obtain in a systematic and reliable fashion and was therefore, not used for the staging of AKI by the KDIGO and ADQI. Third, external validation of our models needs to be performed in the future. Lastly, additional biomarkers are not included in our current models and might help improve identification of patients at risk for AKI and increased mortality [41–43].

## Conclusion

This study demonstrates the importance of reduced renal function and AKI for the long-term survival after LuTx. Findings should help identify patients at risk for AKI and to adjust pre- and post-operative management accordingly. Together with few clinical factors, the continuous parameter d7/preHD-sCr predicts mortality equally well as the commonly used AKI definitions by KDIGO and ADQI and is easier to apply.

## Supporting information

S1 File Anonymized data set with variable description. (XLSX)

S1 Table Multivariable Cox models including postoperative dialysis. (PDF)

## Abbreviations

ADQI: Acute Disease Quality Initiative
AKI: acute kidney injury
CI-95%: 95% confidence interval
CKD: chronic kidney disease
CKD-EPI: Chronic kidney disease epidemiology collaboration
d7: day 7
d7/preHD-sCr: serum creatinine value at day 7 for patients who did not receive hemodialysis, and serum creatinine value immediately before initiation of hemodialysis
ECMO: extracorporeal membrane oxygenation
eGFR: estimated glomerular filtration rate
FEV1: forced expiratory volume in one second
FFP: fresh frozen plasma
FVC: forced vital capacity
HR: hazard ratio
ICU: intensive care unit
IQR: interquartile ranges
KDIGO: Kidney Disease Improving Global Outcomes
LAS: Lung Allocation Score
LuTx: lung transplantation
Max: maximum
Min: minimum
PGD: primary graft dysfunction
pRBC: packed red blood cells
RRT: renal replacement therapy
sCr: serum creatinine
SD: standard deviation
UO: urine output
