# Supplementary material for: Kidney injury after lung transplantation: Long-term mortality predicted by post-operative day-7 serum creatinine and few clinical factors
Source: PLoS One. 2022 Mar 4;17(3):e0265002. doi: 10.1371/journal.pone.0265002 (PMC8896732; doi:10.1371/journal.pone.0265002)
Supplement: S1 Table — (PDF) [file pone.0265002.s002.pdf]

S2 Table – Multivariable Cox models including postoperative dialysis

**d7/preHD-sCr**<br>**Concordance index 0.75 (0.73)**			
	HR	p	CI-95%
Body weight (kg)	1.031	0.019	1.005 – 1-057
>2 transfused platelets intraoperative	2.224	0.025	1.105 – 4.476
>1 transfused platelet postoperative	2.027	0.051	0.997 – 4.119
d7/preHD-sCr (µmol/L)	1.011	< 0.0001	1.006 – 1.017
Dialysis post Tx during first 7 days	3.674	0.057	0.963 – 14.014

Variables for the models were selected as described in the Methods and according to the univariable Cox analyses (Table 2). Concordance index value in brackets indicates the value after 200-fold bootstrapping.

Abbreviations: CI-95%, upper and lower limits of the 95% confidence interval; d7/preHD-sCr, serum creatinine value at day 7 for patients who did not receive hemodialysis, and serum creatinine value immediately before initiation of hemodialysis; HR, hazard ratio; Tx, transplantation.
